# Opioid and gabapentinoid prescriptions in England from 2015 to 2020

**DOI:** 10.1371/journal.pone.0276867

**Published:** 2022-11-28

**Authors:** Yixue Xia, Patrice Forget

**Affiliations:** 1 Epidemiology group, Institute of Applied Health Sciences, School of Medicine, Medical Sciences and Nutrition, University of Aberdeen, Aberdeen, United Kingdom; 2 Anaesthesia department, NHS Grampian, Aberdeen, United Kingdom; 3 The European Society of Anaesthesiology and Intensive Care (ESAIC), Pain AND Opioids after Surgery (PANDOS), Research Group, Brussels, Belgium; University of South Australia, AUSTRALIA

## Abstract

**Purpose:**

Concerns gradually arose about misuse of gabapentinoids (gabapentin and pregabalin), especially when used in combination with opioids. Because it can be a driver of usage, trends in prescribing habits may be interesting to analyse. The aim of this study is to examine the evolution of prescriptions of opioids and gabapentinoids in England from 2015 to 2020 at a regional level.

**Methods:**

This study included data from April 2015 to February 2020, focusing on prescribing data, extracted the OpenPrescribing database. We described the evolution of the prescriptions of opioids and gabapentinoids and calculated their ratios for each month. We used Analyses of Variance (ANOVAs) to compare data between and within regions (over time).

**Results:**

During this period, opioid prescriptions remained stable (from -3.3% to +2.2%/year) and increased for gabapentinoids generally (from +1.5% to +2.2%). The ratio between gabapentinoid to opioid prescriptions increased by more than 20% in 2020 compared to 2015, variably between regions (F(6,406) = [120.2]; P<0.001; LSD Test: P<0.001; ANOVA for repeated measures: P<0.05). In 2019, a decline in the ratio occurred in all regions, but only persisting in the London commissioning region in 2020 (-14.4% in comparison with 2018, 95%CI: -12.8 to -16.3).

**Conclusions:**

Gabapentinoids are increasingly prescribed in England. The ratio of gabapentinoid to opioid prescriptions in England increased from 2015 to 2020. The reclassification of gabapentinoids as controlled drugs, in 2019, may have been associated with a significant reduction, although larger prescribers may have been less influenced.

## Background

Opioid analgesics include alkaloids extracted from opium and synthetic analogues that interact with specific central receptors. Usually, opioids are prescribed to relieve acute pain or pain at the end of life [1]. But little evidence supports a lasting effect on chronic pain. However, the use of opioids is not completely safe. Adverse effects of opioids are related to various factors, such as individual differences, dosage, and drug interactions, which are not specific to drug type and route of administration [2].

Gabapentinoids include pregabalin and gabapentin, both of which were initially cleared for seizures. With opioid use disorder becoming an international public health problem, doctors and patients are looking for alternatives. As a result, more prescriptions for gabapentinoids may have been issued. Even if largely uncertain, it was hypothesised by some that gabapentinoids may help reduce opioids, prevent opioid tolerance, improve the quality of opioid analgesic therapy, and treat anxiety [3]. However, these hypotheses have been, at least partially, rejected [4, 5]. Moreover, in high doses, patients may experience euphoria with gabapentinoids, and withdrawal after abruptly stopping use, including seizures [5–7]. In recent years, the gabapentinoid use disorder has developed rapidly and has gradually become a recognized problem around the world. In patients with substance use disorders, especially those involving opioid use, the abuse may even be more serious [8] and the United Kingdom (UK) classified it as a controlled substance in April 2019 [9]. Additionally, the combined use of gabapentinoids and opioids appears to be associated with specific risks. In patients being prescribed opioids and gabapentinoids concomitantly, there is a substantial increase in the risk of opioid-related death [10, 11]. This concomitant use has been accused of being responsible of differences in opioid-related death rates, between different parts of the UK [12]. A similar debate occurred regarding the concomitant use of benzodiazepines and gabapentinoids [13]. Co-prescribing of benzodiazepines is well described, but there is little description of the co-evolution of the prescribing of opioids and gabapentinoids, except for a recent publication in Scotland [10]. This highlights the need to study regional and national differences.

The aim of this study is to examine the evolution of prescriptions of opioids and gabapentinoids in England from 2015 to 2020 at a regional level.

## Methods

This report is written according to the Strengthening Reporting in Observational Studies (STROBE) guidelines.

### Data sources and preparation

We focused on monthly practice -level data in England from April 2015 to March 2020 and aggregated it at a regional level. In each National Health Service (NHS) Primary Care prescribing organization in the UK, the prescribing data set monthly published by NHS Digital counts for each different drug and dose, describing the number of prescriptions and the total cost. These data come from institutions such as community pharmacies and contain all drugs that have been assigned.

We extracted prescription information from the open OpenPrescribing database for analysis [15]. OpenPrescribing is an online service launched in 2016. Prescribing information is sourced from NHS Digital publishing NHS Business Services Administration monthly and annual prescription data sets and static prescribing trend reports. All data is grouped by drug name and any name available in multiple formulas is combined. OpenPrescribing collects information coming from the following sources: Medications codes and names are also from the NHS Business Service Authority’s Information Portal. Clinical Commissioning Groups (CCGs) and practice prescribing settings, are from NHS Digital’s data downloads (epraccur.csv), used under the terms of the Open Government Licence. CCG names and codes and CCG geographic boundaries are from the Office for National Statistics (ONS) (www.ons.gov.uk). Practice locations are based on data from NHS Digital/ONS.

### Data extraction and classification

We extracted prescription data (opioid analgesics, section 4.7.2 of the formularium) (for a full list, see https://openprescribing.net/bnf/040702/; pregabalin, code 0408010AE; and gabapentin, code 0408010G0) as.csv files. All orders (from April 2015 to February 2020) were compiled according to different regions of England. Capital expenditure was counted per year, covering the annual total of pregabalin and gabapentin prescriptions across all NHS England regional teams.

### Statistical analysis

After compiling the prescriptions data (opioids and gabapentinoids), we calculated the ratios of gabapentinoid (gabapentin and pregabalin) to opioid prescriptions. We displayed these using a line graph with their 95% confidence intervals, calculated for each month, on a rolling year.

The prescription ratios were analysed, after checking the distribution normality using graphical methods, using a one-way Analysis of Variance (ANOVA) to compare prescription counts, and ratios between gabapentinoid and opioid prescriptions, between the different regions, and ANOVAs for repeated measures to compare each region over time. IBM SPSS (SPSS Statistics for Windows, version 25 (SPSS Inc., Chicago, Ill., USA) was used for all the comparison. A P-value <0.05 was considered statistically significant.

### Ethics approval

This study does not require patient consent or ethical approval as the data used is publicly available, anonymous and aggregated at source.

## Results

### Evolution of opioid prescription in England

**Fig 1:**
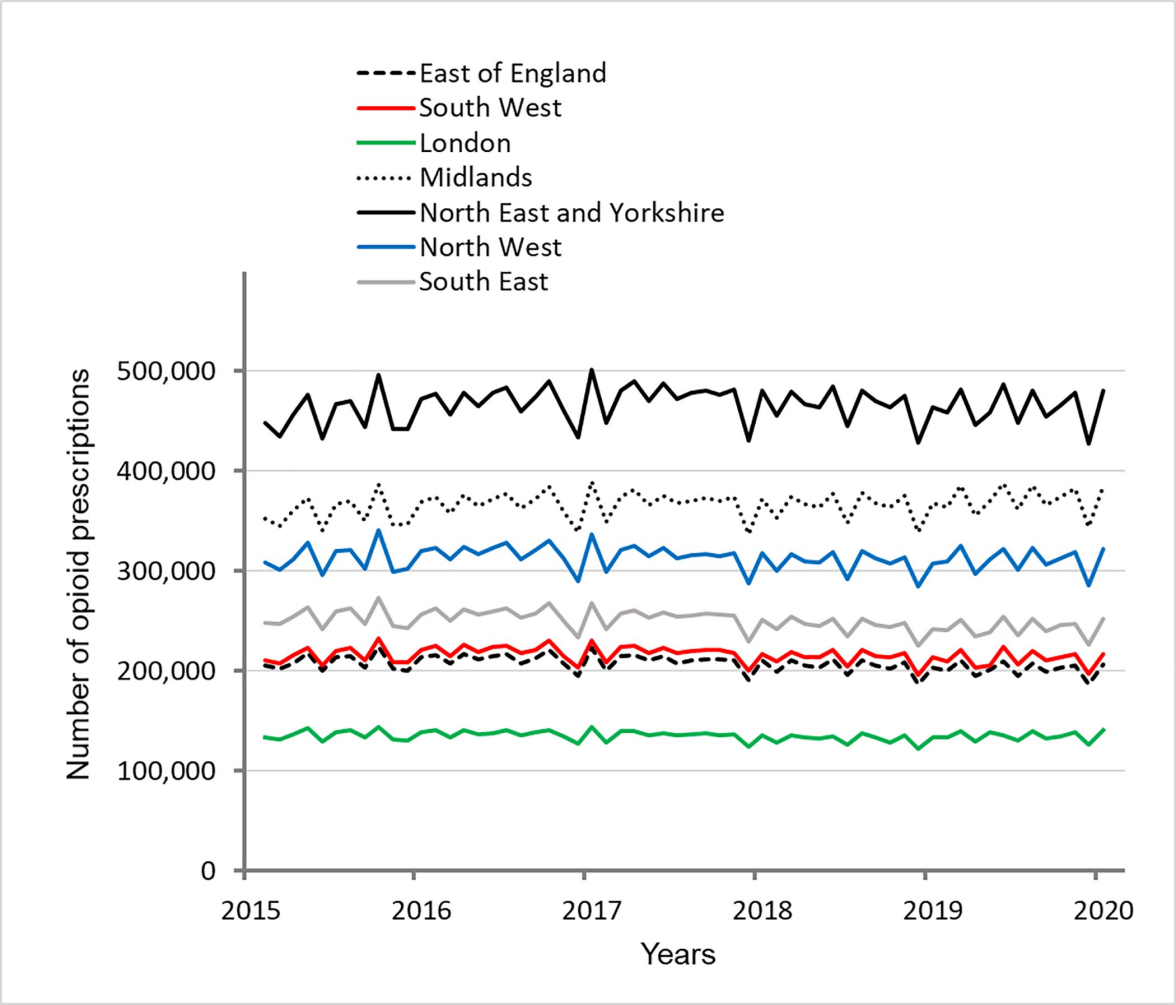
Number of prescriptions for opioid analgesics in each region of England.

Between April 2015 and February 2020, the number of opioid prescriptions in regions of England generally remained stable, ranging from -3.3% to +2.2% of change every year (Fig 1, Table 1).

**Table 1:** Mean monthly prescription count (averaged on the yearly basis) for opioids and gabapentinoids, and their ratio (with 95% confidence intervals– 95%CI–calculated, for each month, on a rolling year) in each region of England.

	Prescriptions	East of England				South West				London				Midlands				North East, Yorkshire				North West				South East			
	(monthly average)																												
**2015**	Opioids	209813				216462				136637				360289				457997				314235				255071			
	Gabapentinoids	99462				87962				88537				160010				185128				157301				112926			
	Gabapentinoid/Opioid ratio (95%CI)	0.474	(0.493	to	0.461)	0.406	(0.419	to	0.393)	0.648	(0.671	to	0.625)	0.444	(0.460	to	0.428)	0.404	(0.416	to	0.392)	0.500	(0.518	to	0.483)	0.443	(0.460	to	0.425)
**2016**	Opioids	211687				220083				137142				366760				468079				317519				256319			
	Gabapentinoids	111169				98127				100452				177724				206198				174201				125536			
	Gabapentinoid/Opioid ratio (95%CI)	0.525	(0.540	to	0.515)	0.446	(0.455	to	0.436)	0.732	(0.750	to	0.714)	0.484	(0.497	to	0.472)	0.440	(0.449	to	0.431)	0.548	(0.562	to	0.535)	0.489	(0.503	to	0.476)
**2017**	Opioids	210213				218992				135921				367791				472951				315138				253914			
	Gabapentinoids	123264				109317				113120				197516				229733				192258				139521			
	Gabapentinoid/Opioid ratio (95%CI)	0.586	(0.596	to	0.581)	0.499	(0.504	to	0.494)	0.832	(0.849	to	0.815)	0.537	(0.545	to	0.528)	0.486	(0.491	to	0.480)	0.610	(0.618	to	0.602)	0.549	(0.559	to	0.540)
**2018**	Opioids	204679				213758				132149				364751				466631				309167				245890			
	Gabapentinoids	132826				115943				123193				215541				247176				206896				149142			
	Gabapentinoid/Opioid ratio (95%CI)	0.649	(0.654	to	0.647)	0.542	(0.545	to	0.540)	0.932	(0.952	to	0.913)	0.591	(0.595	to	0.586)	0.530	(0.532	to	0.528)	0.669	(0.674	to	0.664)	0.607	(0.612	to	0.601)
**2019**	Opioids	201538				211603				133725				369234				461972				309545				242288			
	Gabapentinoids	138967				117964				109657				229790				251415				215364				156517			
	Gabapentinoid/Opioid ratio (95%CI)	0.690	(0.692	to	0.688)	0.557	(0.559	to	0.556)	0.821	(0.824	to	0.817)	0.622	(0.625	to	0.619)	0.544	(0.545	to	0.543)	0.695	(0.698	to	0.693)	0.646	(0.648	to	0.644)
**2020**	Opioids	199369				210202				135237				370295				461678				308719				241610			
	Gabapentinoids	142270				120299				107617				241681				255145				222747				162378			
	Gabapentinoid/Opioid ratio (95%CI)	0.710	(0.711	to	0.710)	0.570	(0.570	to	0.570)	0.797	(0.797	to	0.796)	0.651	(0.651	to	0.650)	0.553	(0.553	to	0.552)	0.720	(0.720	to	0.720)	0.669	(0.669	to	0.668)

### Evolution of gabapentinoid prescription in England

**Fig 2:**
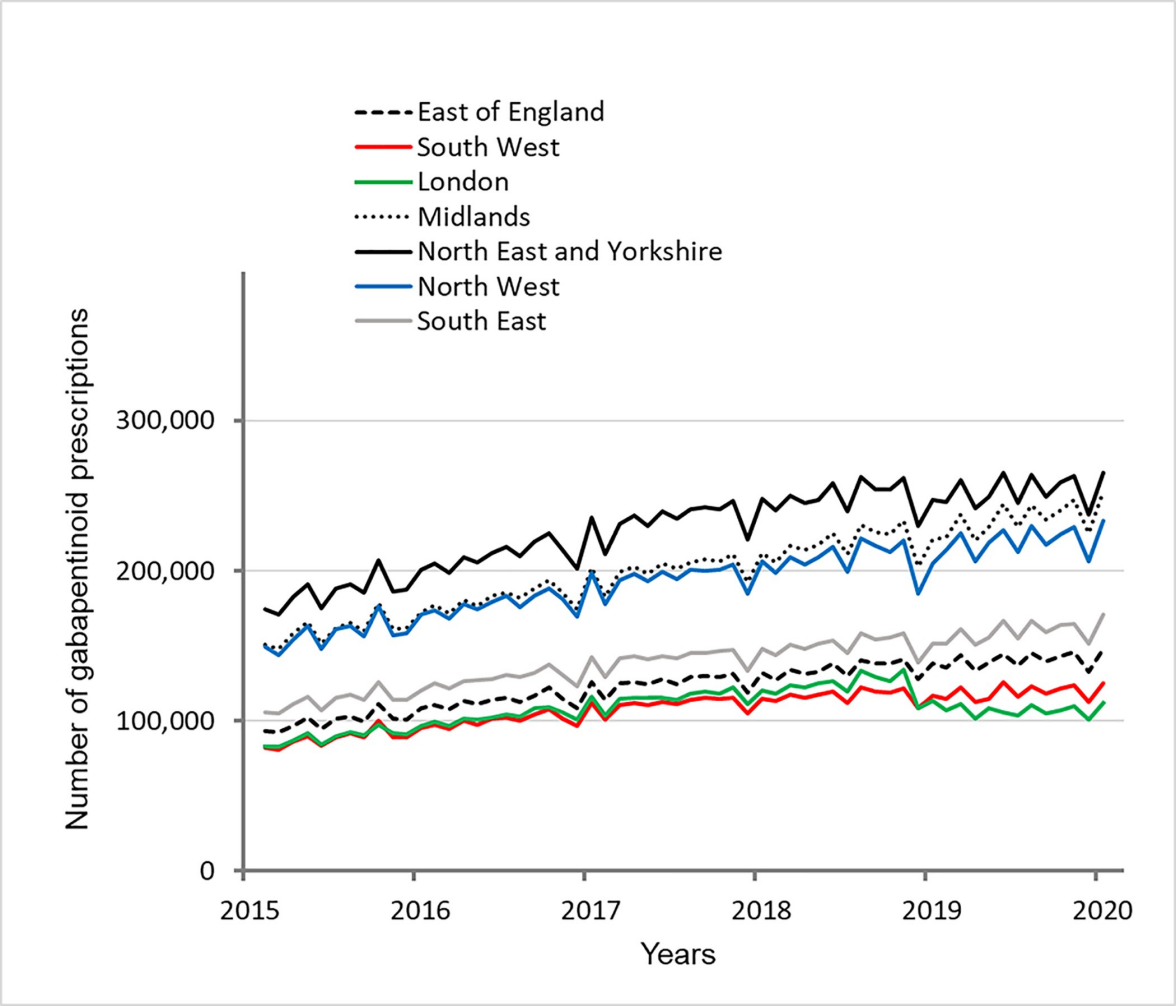
Number of prescriptions for gabapentin and pregabalin in each region of England.

From April 2015 to February 2020, the number of prescriptions of gabapentin and pregabalin in England continuously increased, ranging from +1.5% to 11.9% every year (Fig 2, Table 1).

In early 2019, all regions simultaneously showed a decline, but only persistent in the London commissioning region, falling and remaining 12.3% lower than before.

### Evolution of the ratio between gabapentinoid and opioid prescription in England, between and within regions

To study conjointly the evolution of gabapentinoid and opioid prescription, we calculated the ratio of gabapentin and pregabalin to opioids. A one-way ANOVA was performed to compare the ratios and showed statistically significant difference between regions (F(6,406) = [120.2]; P<0.001; LSD Test: P<0.001).

**Fig 3:**
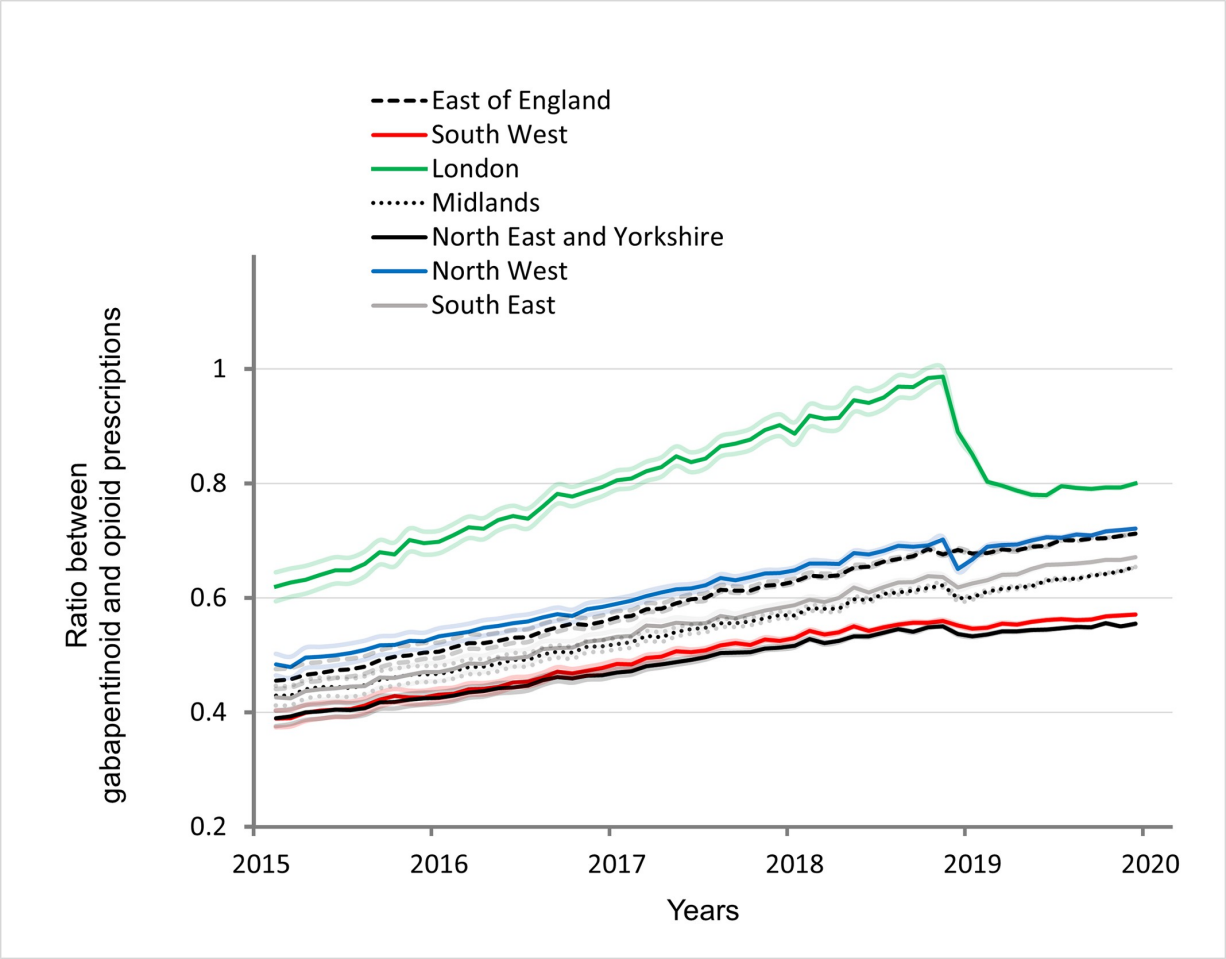
Ratio between gabapentinoid and opioid prescriptions in each region of England. The faded lines represent 95% confidence intervals calculated, for each month, on a rolling year.

Between April 2015 and February 2020, the evolution of the ratio increased overall, with each region experiencing an increase of more than 20% in 2020 compared to 2015 (P<0.05) (Fig 3, Table 1). In 2019, the decrease in gabapentinoid prescriptions was associated with a variably distributed decline in the ratio between gabapentinoid and opioid prescriptions, significant in all regions (ANOVA for repeated measures within regions: P<0.001) but only persisting in the London commissioning region in 2020 (-14.4% in comparison with 2018; 95%CI: -12.8 to -16.3). Costs were explored while considering all prescriptions as a whole, even if a pharmaco-economic study is beyond the purpose of this work. Costs linked to prescriptions increased rapidly from 2015 to 2019, and the London commissioning region increased the most.

## Discussion

### Prescription changes

This study describes the regional evolution in opioid and gabapentinoid prescriptions in England. The number of opioid prescriptions in various regions has essentially stabilized over time (ranging from -3.3% to +2.2%). Meanwhile, prescriptions of gabapentin and pregabalin increased between 2015 and 2020 (ranging from +1.5% to 11.9% every year). Additionally, we found that prescriptions for gabapentin and pregabalin declined in most areas by early 2019, after which the rate of prescription growth declined significantly but remained lower in 2020 only in the London commissioning region (by 12.3%).

In order to study the co-evolution of the prescriptions of gabapentinoids and opioids, we looked at the ratios between gabapentinoids and opioids. From 2015 to the end of 2018, there was a marked increase in all regions, especially in the London commissioning region. At the beginning of 2019, the ratio of the different regions fell, after which the growth rate remained at a low level, but only significantly lower in the London commissioning (-14.4%; 95%CI:-12.8 to -16.3).

### Implications in terms of societal impact and national policy

As the public became aware of the potential risk of opioid use disorder, medical staff began to reconsider pain management and seek more appropriate medication prescriptions, including non-opioid pain relievers [4, 5]. Among other things to reduce opioid dependence, gabapentin and pregabalin have been gradually introduced. Between 2007 and 2017, the proportion of patients receiving primary opioid analgesics in combination with prescriptions of gabapentinoids or opioid analgesics in combination with prescriptions of benzodiazepines in primary care may have tripled approximately [16]. In some series, 20 to 25% of patients who started treatment with gabapentinoids were prescribed opioids [17]. If the goal were to partially prevent opioid tolerance and improve the quality of treatment, especially for neuropathic pain, this effect could have been achieved at the cost of increased side effects, and, at best, uncertain [14, 8–20]. In the treatment of non-neuropathic pain, the use of gabapentinoids in combination with opioid analgesics may reduce short-term opioid use and short-term pain levels, but compared to placebo, the incidence of drug-related adverse reactions is higher [21–23].

Although gabapentinoids were initially identified as unlikely to be abused, in recent years gabapentinoids use disorders has gradually become a public health problem that cannot be ignored [3, 8, 10, 11, 16]. In 2016, the UK’s Advisory Council on the Misuse of Drugs recommended that pregabalin and gabapentin be controlled under the Misuse of Drugs Act 1971 as Class C substances and listed under the Misuse of Drugs Regulations 2001 as Schedule 3 [9]. In the UK, controlled drugs are divided into classes A, B and C, depending on potential harms. Class C drugs are considered the least harmful. Schedule 3 implies specific requirements for the prescribing, dispensing, recording and safe storage of certain drugs, such as certain benzodiazepines. In April 2019, pregabalin and gabapentin were classified as Class C substances and listed as additional substances under the Misuse of Drugs Regulations 2001 [24]. The reclassification decision is based on risk of gabapentinoid misuse, abuse and diversion [9]. The decision appears to be linked to the drop in prescriptions of gabapentin and pregabalin in early 2019 we observed in this study.

It was important for all of us to study these regional differences. A multi-regional analysis was essential to consider as some regions may have more or less success in limiting drug-related problems. Although the reasons for the variability observed (in the present study) are uncertain and beyond our analyses, these data open the way to the development and discussion of different strategies. The observed patterns may also warrant future work aimed at better explaining determinants and outcomes, especially those that are modifiable. Importantly, due to the data we had, only prescriptions from England (but dispensed in the UK), were analysed. It would make sense to supplement these analyses by data from other nations and countries and, at least in the UK, to study the effect of other major guidelines, such as the National Institute for Health and Care Excellence (NICE) guidelines on chronic pain, published in 2021 [25].

### Limitations

Despite the large amount of data representing the entire national prescription rate in England, this work has its limitations. The use of OpenPrescribing data implies that inherent limitations must be considered. These data analyses include primary care only and are not weighted by quantity or strength of items prescribed [15]. This is especially important given the change in gabapentinoid controlled drug scheduling during the study period, limiting the maximum number of days for each prescription. Most importantly, the data has been aggregated, excluding any analysis (and interpretation) at the patient level. This means that the ratios between gabapentinoid and opioid prescriptions have been observed at the regional level and cannot be automatically extrapolated to the patient level. In other words, it cannot be asserted that these data can be considered as a surrogate for the concomitant use of opioids and gabapentinoids. Prescribing patterns may have been different, depending on the practice or over time and it is not possible to describe what proportion of patients are prescribed both these drugs, as this is not available within the data. Importantly, comparisons between regions (for prescriptions numbers) are very difficult to interpret because the number of inhabitants is different too. However, the differences observed and the variability of the ratio were consistently observed and deserve to be underlined, particularly seeing their implications at a Public Health level.

## Conclusions

In England, the number of opioid prescriptions remained stable over the study period (2015 to 2020), but gabapentinoid prescriptions increased. The increase in the ratio between both suggests that gabapentinoids have not replaced opioids in the therapeutic armamentarium.

The decision to reclassify gabapentinoids as drugs at risk of misuse, abuse, and diversion appears to be linked (at least initially) to a decline in gabapentin and pregabalin prescriptions.

## Supporting information

S1 Checklist STROBE statement—checklist of items that should be included in reports of cohort studies. (DOC)

## Abbreviations

ANOVA: Analysis of Variance
CCG: Clinical Commissioning Group
GP: General Practitioner
NHS: National Health Service
SD: Standard deviation
STROBE: Strengthening Reporting in Observational Studies
