# Supplementary material for: Opioid and gabapentinoid prescriptions in England from 2015 to 2020
Source: PLoS One. 2022 Nov 28;17(11):e0276867. doi: 10.1371/journal.pone.0276867 (PMC9704625; doi:10.1371/journal.pone.0276867)
Supplement: Attachment — Submitted filename: Authors_Response2.docx [file pone.0276867.s003.docx]

Dear Editor, dear Reviewers,

Thank you for the kind and constructive comment. You will find here a revised version of our work. We hope that this will encounter all your concerns. We remain, of course, open to any other suggestion.

Kind regards,

Patrice Forget and Yixue Xia

Reviewer #1: Thank you for the revision to this paper.

However, I still have concerns regarding the basis for the analyses. As stated in my previous review, it is not possible to tell from the prescribing data available at OpenPrescribing.net whether a patient is taking opioids and gabapentinoids concomitantly, and that the limitations should be strengthened.

The paper now states "However, the differences observed and the variability of the ratio were consistently observed and deserve to be underlined, particularly seeing their implications at a Public Health level", and now includes "we described the evolution of the prescriptions of opioids and gabapentinoids, and calculated their ratios (as a surrogate for their concomitant use at a population level) for each month."

I do not believe you can use this data to show any sort of surrogate of concomitant taking of opioid and gabapentinoid, for reasons stated above, and therefore this should be revised to remove this suggestion.

Answer: Thank you for the comment. This is right, and what we may believe is different than what we can state. In other words, we fully agree with the reviewer that this is factually not supported by the data presented and can in no way be taken as certain. Accordingly, we have checked, throughout, and removed any mention of "surrogate" or “concomitant” and strengthened the limitation section as follows:

*This means that the ratios between gabapentinoid and opioid prescriptions have been observed at the regional level and cannot be automatically extrapolated to the patient level. In other words, it cannot be asserted that these data can be considered as a surrogate for the concomitant use of opioids and gabapentinoids. Prescribing patterns may have been different, depending on the practice or over time and it is not possible to describe what proportion of patients are prescribed both these drugs, as this is not available within the data.*
